# Association between the 24-hour movement guidelines and executive function among Chinese children

**DOI:** 10.1186/s12889-022-13420-5

**Published:** 2022-05-20

**Authors:** Xia Zeng, Li Cai, Wenhan Yang, Weiqing Tan, Wendy Huang, Yajun Chen

**Affiliations:** 1 School of Public Health, Guangdong Pharmaceutical University, Guangzhou, 510310 China; 2 School of Public Health, Sun Yat-Sen University, Guangzhou, 510080 China; 3 Guangdong Provincial Engineering Research Center of Public Health Detection and Assessment, Guangdong Pharmaceutical University, Guangzhou, 510310 China; 4 Health Promotion Center for Primary and Secondary Schools of Guangzhou Municipality, Guangzhou, 510145 China; 5 Department of Sport and Physical Education, Hong Kong Baptist University, Kowloon Tong, Hong Kong, China

**Keywords:** Children, Executive function, The 24-h movement guidelines

## Abstract

**Objective:**

Childhood is a critical period for brain development. However, it remains unknown whether the behaviors in a typical 24-h day are related to children’s executive function (EF). This study aimed to investigate the relationship between the 24-h movement guidelines and children’s EF.

**Method:**

Children aged 7–12 years (*n* = 376) were studied in 2017 in China. Physical activity (PA) was accelerometer-derived, while screen time (ST) and sleep duration were self-reported. Meeting the 24-h movement guidelines was defined as: 1) ≥ 60 min/day of moderate-to-vigorous PA; 2) ≤ 2 h/day of recreational ST; 3) 9–11 h/night of sleep. EF was assessed by the Wisconsin Card Sorting Test (WCST). Number of completed categories (CC), shifting efficiency (SE), non-perseverative errors (NPE), and failure to maintain set (FMS) were used to measure four processes of EF, respectively represented global performance, cognitive flexibility, efficiency in rule discovery, and sustained attention. Generalized linear mixed models (GLMM) were completed to explore the associations of meeting the PA, ST, and sleep duration recommendations with four processes of EF.

**Results:**

Statistically significant positive associations were observed between the number of guidelines met, regarded as a continuous variable, with CC [*β* = 0.343 (95% confidence interval [CI]: 0.125, 0.561)] and SE [*β* = 4.028 (95% CI: 0.328, 7.727)], while number of guidelines met negatively related to NPE [*β* =  − 4.377 (95% CI:-7.952,-0.802)]. Participants not meeting the two recommendations for PA and sleep duration had lower scores in CC [*β* = -0.636(95% CI:-1.125,-0.147)] and SE [*β* = -10.610 (95% CI:-18.794,-2.425)] compared with those meeting the two, suggesting inferior global performance and worse efficiency in rule discovery. However, ST recommendation had no significant association with any processes of EF.

**Conclusion:**

Meeting more recommendations of the 24-h movement guidelines was associated with superior EF in children. Specifically, more PA and healthy sleep duration should be encouraged to promote children’s EF.

**Supplementary Information:**

The online version contains supplementary material available at 10.1186/s12889-022-13420-5.

## Background

Executive function (EF) is a multifaceted and multidimensional cognitive domain in which several underlying processes—such as planning, working memory, sustained attention, integration and feedback—coordinate together to perform both current and future goal-directed behaviors [1]. The Wisconsin Card Sorting Test (WCST) is regarded as “the gold standard of EF task” because of a highly sensitive indicator for cognitive flexibility, planning, and set maintenance [2]. Evidence suggests that EF early in life appears to be quite predictive of achievement and health throughout life [3]. For example, Moffitt et al. found that children who at ages 3 to 11 had better EF were more likely as teenagers to still be in school and were less likely to make risky choices in future, such as smoking, drugs use, etc. [3], which shows that the development of EF in childhood deserves more attention. A child’s EF development is affected by cultural and environmental factors including education, diet, environmental exposures, and daily movement behaviors [4, 5]. Among them, daily movement behaviors are considered to be important modifiable factors to promote children’s EF [4].

Achieving high levels of physical activity (PA), low levels of recreational screen time (ST), and enough sleep has been individually positively associated with children’s EF development [6–8]. Preadolescent children with higher PA tended to have better EF performance compared to their peers with less PA [6]. Excessive ST were reported independently related to weak inhibitory control, indicating that the more time children spent on screen, the worse their EF [7, 9]. In addition, sleep deprivation led to worse working memory, speed, and accuracy, and negatively affected the brain’s prefrontal cortex, leading to executive dysfunction [10]. However, the fact that PA, ST and sleep duration have been separately from each other is concerning, because research has shown that these three behaviors are codependent and should be considered simultaneously [11].

The 24-h movement guidelines represent a shift from focusing on certain individual movement types to the all-day behavior model. According to the latest data, only between 3 and 10% of children and adolescents from different countries around the world met all three recommendations [12–14]. Previous studies looking at the 24-h movement guidelines and health indicators mostly focused on examining associations between the combinations of PA, ST, and sleep duration with physical health outcomes [15–17]. To our knowledge, only 10 studies have reported the relationship between 24-h movement guidelines and mental health indicators, and one of the 10 had EF as an outcome variable [18, 19]. However, the above-mentioned study [19] conducted among children aged 9–10 years evaluated EF by using scales instead of the WCST, and it did not consider adjustment for Intelligence Quotient (IQ), which is recognized as a factor closely related to EF and might play a role in the association between the 24-h movement guidelines and EF [20]. In conclusion, little is known about the extent of the gaps in the current literature regarding the 24-h movement guidelines in relation to children’s EF. It is also unclear whether some combinations of PA, ST and sleep duration are more strongly associated with EF.

To address the pending evidence gaps, we examined if meeting the 24-h movement guidelines relate to EF while adjusting for IQ. It was hypothesized that children meeting all three recommendations of the 24-h movement behaviors would have superior EF compared to those who meeting two, one, or none of the recommendations.

## Methods

### Study design and participants

This study was conducted in 2017 using data from a school-based prospective cohort study’s baseline examination (Registration number: NCT03582709). The study complied with Declaration of Helsinki and was approved by the Ethics and Human Subject Committee of Sun Yat-sen University. Design of this study has been described elsewhere [21]. Briefly, we performed a two-stage cluster sampling strategy to recruit participants. First, we randomly selected five districts including three urban areas (Yuexiu District, Tianhe District, Liwan District) and two suburban areas (Panyu District, Huangpu District) in Guangzhou, a city located in southern China. Second, we randomly selected one primary school within every district. A brief meeting was arranged for school teachers to facilitate the implementation of this project. Informed consent for the study was distributed to each child. Children were advised to discuss with their parents and returned the parents’ signed informed consent to the school if they and their parents were willing to participant. Subsequently, 637 students of five schools with written informed consent were enrolled in this study.

### Summary of all the measurements

The 24-h movement guidelines, as exposure variable were synthesized by PA, ST and sleep duration. PA was objectively measured by ActiGraph GT3X accelerometer, ST and sleep duration were collected by questionnaires with good reliability and validity [22]. We assessed EF using the WCST, which is often used to assess EF performance in children [23].

### Exposures

#### Physical activity

 PA was measured using ActiGraph GT3X accelerometer (ActiGraph, Pensacola, Florida, USA), one of the most commonly used activity monitors in children [24]. Accelerometers were attached to an elastic belt and worn above the iliac crest on the right side. Meanwhile, children were asked to fill in a PA log with the help of their parents, providing detailed information of PA and sedentary behaviors corresponding to the accelerometer records. All children were instructed to wear the devices during waking hours for 7 consecutive days, except during water-based activities (swimming and bathing). Sampled at 30 Hz, data were collected starting at 6:00 am, and ended at 11:59 pm using the unit of counts per minute (cpm). The accelerometer data files were reintegrated to 30-s epochs for its good sensitivity to detect child's activities, and non-wear periods were identified (and excluded from further analysis) by scanning the data array for periods of at least 60 min of consecutive zeros (allowing for 2 min of non-zero interruptions) [24]. We limited our analyses to participants who wore the device for ≥ 10 h/day for ≥ 4 days (including 3 weekdays and 1 weekend) according to the software (ActiLife V.6.13.3). Prediction equations were used to identify cut-points for classifying activity into sedentary time (< 100 cpm), light-intensity (100-2295 cpm), moderate-intensity (2296-4011 cpm) and vigorous-intensity (≥ 4012 cpm) PA [25]. Moderate-to-vigorous-intensity PA was calculated as the sum of moderate-intensity and vigorous-intensity PA. Total wear time was recorded and averaged per day.

#### Screen time and sleep duration

 Under the assistance of the parent, children were asked to report the average amount of time (h and min) spent daily on various recreational screen-based activities (e.g., watching television or videos, playing video games, using the computer) in the past 7 days. Children’s bedtimes, waketimes, sleep latency, and nap duration over the previous week were recorded separately for weekdays and weekends. Sleep latency was measured by asking “how long it usually takes you to fall asleep after you go to bed?” The sleep duration was calculated as follows: sleep duration (hours) = (waketimes − bedtimes) − sleep latency; weekly sleep duration (hours) = [5 × (weekday sleep duration) + 2 × (weekend sleep duration)]/7. Questionnaires, used to collect ST and sleep duration, have been proven to have a good internal consistency with the coefficient was 0.80 and 0.74 and test–retest reliability with the coefficient was 0.59 and 0.63 in ST and sleep duration, respectively [22].

#### 24-h movement guidelines

 Children who reported moderate-to-vigorous-intensity PA ≥ 60 min/day, accumulating ≤ 2 h of daily recreational ST, and sleeping 9–11 h/night were considered to be meeting all three recommendations of the 24-h movement behaviors [26].

### Outcomes

#### Executive function (WCST)

The WCST represents a widely used neuropsychological test for EF assessment [27]. We used 128-cards WCST’s computerized version to examine children’s EF. Participants were asked to sort cards according to one of the three rules (color, shape and number) by mouse clicking [27]. Four WCST indices were used for analysis: number of completed categories (CC), shifting efficiency (SE), non-perseverative errors (NPE), and failure to maintain set (FMS) [28, 29]. CC was the number of sets of 10 consecutive correct responses. NPE comprised all errors except perseverative errors. FMS was the number of incorrect responses after 2–9 consecutive correct responses. SE was calculated as proposed by [29]: SE score = CC*6 + [128 − (the number of cards used)]. This scoring method takes efficiency in achieving CC into account by rewarding unused cards, which helps to detect differences in task performance. The CC measure gives a global performance score, and higher CC score indicates superior global performance. Higher SE score suggests better cognitive flexibility. Lower NPE and FMS score are related to higher efficiency in rule discovery and less difficulty in sustained attention.

### Covariates

Children’s birth date, sex, paternal and maternal educational level, and household monthly income were collected by questionnaires filled out by the parents. Height and weight were measured by research assistants. Body mass index (BMI) was calculated by dividing body weight (kg) by height squared (m^2^). Children’s IQ was initially assessed using the Combined Raven’s Test (second edition). With the advantages of non-verbal and less affected by language and ethnic differences, this test has widely used in China, especially for school-aged children [30]. Through the group test, every child received a booklet and a sheet of answer paper in the quiet classrooms. Under trained research assistants’ guidance, participants had 40 min to complete this test.

### Statistical analyses

The participants’ descriptive statistics were used to characterize the study population. Mean ± standard deviation (SD) and sample number (percentage) were presented for continuous and categorical variables, respectively. T-tests and chi-squared tests or Kruskal–Wallis tests were used to compare sex differences among continuous and categorical variables. Descriptive statistics for the proportion of participants meeting different combinations of recommendations are presented in Fig. 2.

Analysis of covariance (ANCOVA) was conducted to examine group differences in meeting different number of guidelines with the WCST’s four indices (CC, SE, NPE, FMS). A trend analysis was completed to examine whether there was a gradient of meeting more recommendations with higher or lower scores among EF’s four processes. Generalized linear mixed models (GLMM) were completed to explore the associations of meeting or not meeting different combinations of recommendations with WCST’s indices (CC, SE, NPE, and FMS) after controlling for sex, age, paternal/maternal educational level, household monthly income, BMI, and IQ. Schools were fitted as random effects in models. The results were reported with unstandardized path coefficients (*β*), and 95% confidence interval (CI). All analyses were conducted using SPSS 21.0 (IBM, Armonk, NY, USA). We defined statistical significance as *P* < 0.05 for a two-tailed test.

## Results

### Participants characteristics and adherence to movement behavior recommendations

**Fig. 1:**
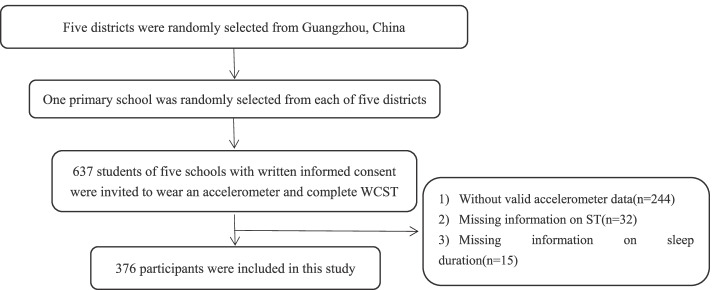
Flow diagram of participant selection and assignment

**Fig. 2:**
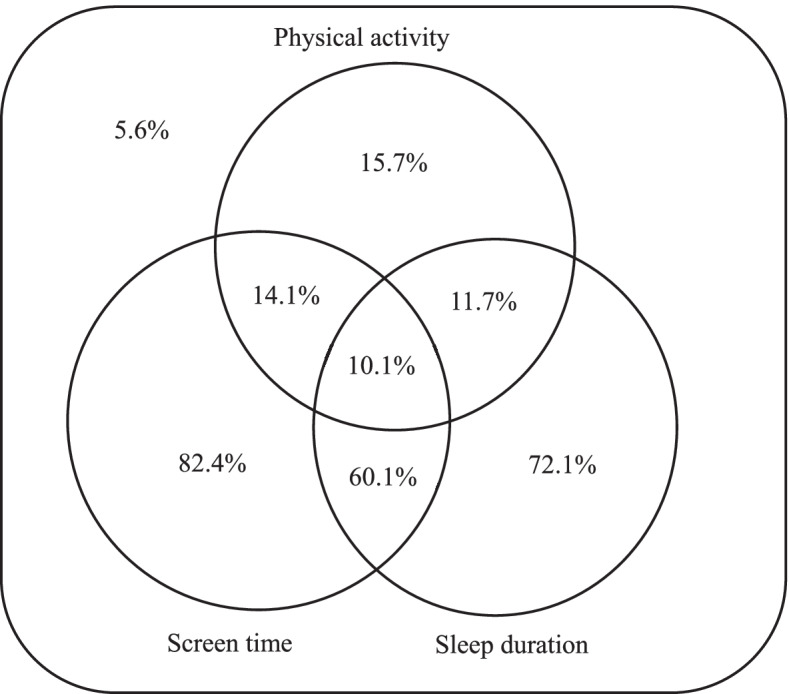
Venn diagram showing the proportion (%) of participants meeting no guidelines, physical activity, screen time, and sleep duration recommendations, and combinations of these recommendations in the full study sample (*N* = 376)

A total of 637 children were enrolled in this study, and 376 of them had valid data and were included in the analyses. Flow diagram of participants selection was showed in Fig. 1. Participants’ demographics and characteristics stratified by sex are summarized in Table 1. Three hundred seventy-six children (195 boys [51.9%] and 181 girls [48.1%]; mean [SD] age, 9.17 [1.61] years) completed the questionnaire information and effective wearing of accelerometer to assess PA, and the WCST. Sample’s average time of moderate-to-vigorous-intensity PA was 42.05 ± 16.79 min/day, and boys did more moderate-to-vigorous-intensity PA than girls per day (49.05 ± 16.22 vs. 34.51 ± 13.92, *P* < 0.001). On average (mean ± SD), participants reported spending 0.99 ± 1.12 h/day on ST and 9.35 ± 0.71 h/night on sleep. Overall, 15.7%, 82.4%, and 72.1% of participants met the PA, ST, and sleep duration recommendations, respectively (Fig. 2). Only approximately 10% of participants met all three guidelines. (Table 1 and Fig. 2). We also analyzed differences in demographic characteristics of the analyzed subjects and sampling populations. There were no statistically significant differences between the two groups in age, sex, paternal and maternal educational level, household monthly income, BMI, and IQ (*P* > 0.05), indicating that the included samples were representative of the total sample (Table S1).

**Table 1:** Descriptive characteristics of the participants

	Total (*N* = 376)	Boys (*N* = 195)	Girls (*N* = 181)	*P*
Age (years)	9.17 ± 1.61	9.14 ± 1.60	9.21 ± 1.62	0.667
Paternal educational level				0.426
High school or below	91 (24.6)	42 (21.9)	49 (24.6)	
Junior college	88 (23.8)	46 (24.0)	42 (23.6)	
College or above	191 (51.6)	104 (54.2)	87 (48.9)	
Maternal educational level				0.289
High school or below	87 (23.5)	50 (26.0)	37 (20.8)	
Junior college	104 (28.1)	48 (25.0)	56 (31.5)	
College or above	179 (48.4)	94 (49.0)	85 (47.8)	
Household monthly income				0.989
< 5000, RMB	92 (24.5)	47 (24.1)	45 (24.9)	
5000 ~ 7999, RMB	86 (22.9)	45 (23.1)	41 (22.7)	
8000 ~ 11,999, RMB	55 (14.6)	27 (13.8)	28 (15.5)	
≥ 12,000, RMB	76 (20.2)	40 (20.5)	36 (19.9)	
No answer	67 (17.8)	36 (18.5)	31 (17.1)	
BMI	17.33 ± 7.53	17.57 ± 3.05	17.07 ± 10.40	0.529
IQ score	112.32 ± 16.92	111.46 ± 16.90	113.22 ± 16.94	0.758
LPA (min/day)	261.88 ± 57.64	248.02 ± 54.45	274.75 ± 57.67	** < 0.001**
MPA (min/day)	31.34 ± 11.45	36.09 ± 11.10	26.21 ± 9.45	** < 0.001**
VPA (min/day)	10.71 ± 6.81	12.96 ± 6.65	8.30 ± 6.14	** < 0.001**
MVPA (min/day)	42.05 ± 16.79	49.05 ± 16.22	34.51 ± 13.92	** < 0.001**
Screen time (h/day)	0.99 ± 1.12	0.97 ± 1.09	1.02 ± 1.17	0.669
Sleep time (h/day)	9.35 ± 0.71	9.40 ± 0.75	9.30 ± 0.66	0.178
Number of guidelines met				** < 0.001**
Three	38 (10.1)	32 (16.4)	6 (3.3)	
Two out of three	209 (55.6)	101 (51.8)	108 (28.7)	
One out of three	108 (28.7)	55 (28.2)	53 (29.3)	
None	21 (5.6)	7 (3.6)	14 (7.7)	

Data were presented as Mean ± SD or n (%). *BMI* Body Mass Index, *IQ* Intelligence Quotient, *LPA* light-intensity Physical Activity, *MPA* moderate-intensity Physical Activity, *VPA* vigorous-intensity Physical Activity, *MVPA* Moderate-to-vigorous-intensity Physical Activity

### Associations between meeting the 24-h movement guidelines and executive function

ANCOVAs showed that children meeting more recommendations had higher CC and SE scores and lower NPE scores, indicating superior global performance, better cognitive flexibility, and greater efficiency in rule discovery, respectively (*P*_*for trend*_ <0.05). We also compared sex differences in WCST performance across the four groups, and found that the above statistically significant relationship was only observed in boys, but not in girls. However, this trend did not appear in patterns of recommendations and FMS score for both boys and girls (Table 2).

**Table 2:** Performance of WCST among Four Groups

Variables	Groups (Mean ± SD)				*P* for trend
	None (*n* = 21)	One out of three (*n* = 108)	Two out of three (*n* = 209)	Three (*n* = 38)	
All					
CC	4.43 ± 1.50	4.71 ± 1.67	4.91 ± 1.53	5.66 ± 0.58	**0.002**
SE	43.95 ± 24.27	43.86 ± 26.43	45.74 ± 25.59	57.08 ± 22.02	**0.050**
NPE	43.52 ± 27.02	36.88 ± 25.55	34.56 ± 24.19	27.16 ± 21.60	**0.011**
FMS	1.10 ± 0.83	1.41 ± 1.29	1.42 ± 1.30	1.16 ± 0.97	0.850
Boys					
CC	4.57 ± 1.62	4.82 ± 1.59	5.01 ± 1.40	5.69 ± 0.59	**0.005**
SE	47.86 ± 27.24	45.13 ± 26.33	46.65 ± 25.24	57.63 ± 20.70	**0.072**
NPE	50.71 ± 28.03	35.83 ± 25.16	33.48 ± 23.02	27.75 ± 22.36	**0.029**
FMS	0.86 ± 0.69	1.43 ± 1.26	1.41 ± 1.33	1.13 ± 0.98	0.451
Girls					
CC	4.36 ± 1.50	4.60 ± 1.76	4.80 ± 1.65	5.50 ± 0.55	0.137
SE	42.00 ± 23.49	42.48 ± 26.73	44.84 ± 26.03	54.17 ± 30.31	0.315
NPE	39.93 ± 26.81	38.00 ± 26.17	35.63 ± 25.37	24.00 ± 18.42	0.185
FMS	1.21 ± 0.89	1.38 ± 1.32	1.42 ± 1.28	1.33 ± 1.03	0.675

Data were presented as Mean ± SD. Analysis of covariances were used to detect indices of WCST differences among the four groups

*WCST* the Wisconsin Card Sorting Test, *SD* Standard deviation

*CC* number of Completed Categories. *SE* Shifting Efficiency. *NPE* Non-Preservative Errors. *FMS* Failure to Maintain Set

We further performed GLMM to explore the associations between patterns of meeting various movement behavior recommendations and the WCST’s four indices (Table 3). Statistically significant positive associations were observed the number of guidelines met, regarded as a continuous variable, with CC [*β* = 0.343 (95% confidence interval [CI]: 0.125, 0.561)] and SE [*β* = 4.028 (95% CI: 0.328, 7.727)], while number of guidelines met negatively related to NPE [*β* =  − 4.377(95% CI:-7.952,-0.802)]. Not meeting any recommendations was associated with significantly lower CC score, lower SE score, and higher NPE score than meeting all three recommendations as a categorical variable, respectively implied inferior global performance, worse cognitive flexibility, and lower efficiency in rule discovery (*P* < 0.05).

**Table 3:** Associations between meeting the physical activity, screen time, and sleep duration recommendations and four dimensions of WCST

		*β* (95% CI)		
Guidelines	CC	SE	NPE	FMS
Number of guidelines met	0.343 (0.125,0.561) *	4.028 (0.328,7.727) *	-4.377 (-7.952, -0.802) *	0.032 (-0.155,0.219)
PA				
Meet	Reference	Reference	Reference	Reference
Do not meet	-0.497 (-0.939, -0.055) *	-6.189 (-13.609,1.231)	4.533 (-2.699,11.765)	0.210 (-0.166,0.586)
ST				
Meet	Reference	Reference	Reference	Reference
Do not meet	-0.085 (-0.506,0.335)	-2.198 (-9.177,4.780)	2.162 (-4.671,8.994)	-0.046 (-0.403,0.311)
Sleep Duration				
Meet	Reference	Reference	Reference	Reference
Do not meet	-0.528 (-0.890, -0.166) *	-4.903 (-10.928,1.122)	7.555 (1.651,13.458) *	-0.245 (-0.552,0.063)
PA + ST				
Meet	Reference	Reference	Reference	Reference
Do not meet	-0.643 (-1.101, -0.185) *	-7.015 (-14.710,0.680)	6.261 (-1.239,13.761)	0.133 (-0.259,0.525)
PA + Sleep Duration				
Meet	Reference	Reference	Reference	Reference
Do not meet	-0.636 (-1.125, -0.147) *	-10.610 (-18.794, -2.425) *	6.722 (-1.279,14.723)	0.328 (-0.085,0.741)
ST + Sleep Duration				
Meet	Reference	Reference	Reference	Reference
Do not meet	-0.411 (-0.741, -0.080) *	-5.297 (-10.790,0.196)	6.198 (0.814,11.582) *	-0.109 (-0.404,0.186)
All three recommendations				
Meet	Reference	Reference	Reference	Reference
Do not meet	-0.850 (-1.361, -0.339) **	-12.315 (-20.955, -3.674) *	9.282 (0.830,17.734) *	0.250 (-0.188,0.689)

Model was adjusted by sex, age, paternal/maternal educational level, household monthly income, Body Mass Index and Intelligence Quotient

Schools were fitted as random effects in models

*WCST* the Wisconsin Card Sorting Test, *PA* Physical activity, *ST* Screen time;

*CC* number of Completed Categories, *SE* Shifting Efficiency, *NPE* Non-Preservative Errors, *FMS* Failure to Maintain Set

^*^ *P* < 0.05

^**^ *P* < 0.001

Children not meeting the PA recommendation had a significantly lower CC score [*β* =  − 0.497 (95% *CI*: − 0.939, − 0.055)] than those meeting them. Moreover, not meeting sleep duration recommendations had detectable associations with inferior global performance score and worse efficiency in rule discovery than meeting sleep duration recommendations (*P* < 0.05). No significant associations were found between ST recommendation and WCST’s indices. For specific combinations of recommendations, there were significantly lower CC scores in children who did not meet PA + ST [*β* =  − 0.643 (95% *CI*: − 1.101, − 0.185)], or PA + sleep duration [*β* =  − 0.636 (95% *CI*: − 1.125, − 0.147)], or ST + sleep duration [*β* =  − 0.411 (95% *CI*: − 0.741, − 0.080)] than in children meeting those patterns of the above recommendations. A similar association was observed between meeting recommendations of PA + sleep duration and SE score. Compared with those who met the ST + sleep duration recommendations, participants not meeting those two recommendations had worse efficiency in rule discovery [*β* = 6.198 (95% *CI*: 0.814,11.582)]. No significant association was observed between any individual or concurrent recommendations and FMS score (*P* > 0.05).

## Discussion

To the best of our knowledge, this is the first study to examine the association of 24-h movement guidelines with EF adjusting the analysis for IQ among children aged 6–12 years. The main finding was that children meeting more 24-h movement guidelines’ recommendations had superior EF regarding global performance, cognitive flexibility, and efficiency in rule discovery. Particularly, this pattern was evident for individual and concurrent associations of PA and sleep duration with children’s EF.

Across CC, SE, and NPE, we found that meeting fewer recommendations was associated with worse global performance, cognitive flexibility, and efficiency in rule discovery in a gradient pattern. Consistent with this study’s findings, Walsh et al. [19] observed that children aged 9–10 years who met fewer 24-h movement guidelines’ recommendations had lower global cognition scores using by scale. Similarly, using a parent-reported Child Behavior Checklist, one Canadian study reported that meeting more recommendations of the 24-h movement guidelines was associated with fewer behavioral and emotional problems at 3 years [31]. Another study also reported that meeting none, one, and two recommendations was related to higher difficulties score compared to meeting all three recommendations [32]. Here, further results from GLMM suggested associations between three of the WCST’s indices (CC, SE, and NPE) and patterns of meeting various movement behavior recommendations after adjusting for sex, age, paternal and maternal educational level, household monthly income, BMI, and even IQ. Moreover, we found sex differences in the relationship between 24-h movement guidelines and children's EF, possibly due to the fact that boys participated in MVPA more time than girls in this study.

In particular, evident individual and combined associations of PA and sleep duration with children’s EF were found. Meeting the PA recommendation (alone or in combination with meeting the sleep duration recommendation) was positively related to CC and/or SE scores, indicating better global performance and/or cognitive flexibility. A recent well-documented meta-analysis also provided evidence of PA’s positive effects on EF, attention, and academic performance children (aged 6–12 years) [6]. Additionally, emerging evidence shows that a single exercise and regular participation in PA benefit EF, including memory, attention, and inhibition [33]. The mechanisms that benefit from PA may include increased cerebral blood flow and metabolism, enhanced functional coupling between the brain networks and the provision of neurotrophins, and better neurotransmitter regulation [34].

Another important finding was that participants not meeting sleep duration recommendation had statistically detectable associations with inferior global performance and efficiency in rule discovery compared to those meeting sleep duration recommendation. These results further support Short, et al.’s study, which showed that sleep plays an important role in brain development and plasticity, and greater sleep quality and quantity were positively associated with cognition in children [35]. Sleep deprivation can result in impairments in the brain structure among children, and further lead to executive dysfunction [10]. Thus, our results are consistent with an increasing body of literature suggesting that meeting the sleep duration recommendation (alone or in combination with meeting the PA or ST recommendations) was favorably associated with some indices of WCST. A randomized controlled trial confirmed that increased PA can improve sleep and mood outcomes [36]. Furthermore, one cross-sectional study also reported that youth who regularly meet sleep duration guideline are more physically active [37]. These results converge with those of our study. Our findings highlight that meeting the recommendations of PA and sleep duration can provide unique benefits to CC and SE compared with not meeting the two.

Interestingly, we found that not meeting the ST recommendation was not significantly associated with any indices of the WCST compared to meeting ST recommendation, consistent with previous studies’ finding [38, 39]. Previous studies showed that the associations between ST and mental health outcomes were very small [38, 39]. On the other hand, in the 2016 PAFCTYS, 63.2% of Chinese children aged 9–17 met the ST recommendation [40], which is far lower than 82.4% in this study. This difference in proportion, in addition to different age composition of samples, also suggested that the level of ST may be underestimated in our study because of not including all types of ST, such as the time spent using mobile phones and iPads, which is also a possible reason for the insignificant relationship between ST and children’s EF in present study.

However, the association between ST and mental health has been controversial. Temperate engagement in ST may not lead to behavioral or emotional problems. For instance, previous research found that ≤ 2 h/day of ST was linked to superior global cognition [19]. In contrast, frequent use of video games was related to conduct problems, and increased television viewing was negatively associated with children’s cognitive development [41]. Growing evidence suggests that screen use may differentially impact EF resulting from screen type, content, and task requirements [7]. Results of an interventional study showed that children were more likely to delay gratification after playing an educational app than after viewing a cartoon, and children’s working memory also improved after playing the educational app [42]. One study even pointed out that the adverse effects of ST on EF may due to the occupation or replacement of other activities (such as PA, reading, etc.) that were beneficial to the development of children's EF [43]. Here, ST + PA or ST + sleep duration, rather than individual ST, had statistical correlation with WCST’s certain indices. We speculate that the concurrent associations of ST + PA or ST + sleep duration with children’s EF were mainly driven by PA or sleep duration. Therefore, understanding the multiple dimensions of screen use and its place in modern life may be critical to envision the role of screen use in children.

This study’s key strengths are PA’s objective measurement, and the EF assessment using WCST. Additionally, models were adjusted for several key potential confounders, including IQ and BMI. Although IQ has been proven to be an important factor closely related to children’s EF [20], few studies have considered it as a confounding factor. However, our study’s limitations need to be noted. First, our results are based on cross-sectional data, which do not allow for tracking the durability and consistency of movement behavior adherence over time, precluding any causal inferences, prospective cohort study or randomized controlled study in the future would be need to establish that causality inference. Second, self-reported exposures on ST and sleep duration, even though they were collected from questionnaires with good reliability and validity [22], make our study susceptible to recall and social desirability biases. Third, assessment of ST in this study is not comprehensive, such as excluding time spent on mobile electronic devices, and future research should take them and other newly emerging electric devices into account. Forth, though we did a sensitivity analysis for missing samples, a large proportion of missing data may still affect the results to some extent. Fifth, small sample size of some groups may limit the power to detect associations, which is also an important reason for limiting our analysis of subgroups by sex.

## Conclusions

Meeting more 24-h movement guidelines’ recommendations was associated with superior EF performance in children. As only 10.1% of the sample met all three recommendations, these guidelines’ adoption should be promoted. Additionally, future work should further explore longitudinal data to more concretely decipher these associations’ temporality and intensity.

## Supplementary Information

**Additional file 1: Table S1. **Demographic characteristics of the analyzed subjects and sampling populations. **Table S2. **Performance of WCST with or without meeting recommendations for PA, ST, and sleep duration. **Table S3. **Associations between meeting the physical activity, screen time, and sleep duration recommendations and four dimensions of WCST in boys.

## Data Availability

The data that support the findings of this study are available on request from the corresponding author. The data used during the current study is not publicly available due to privacy or ethical restrictions.

## Abbreviations

EF: Executive function
PA: Physical activity
ST: Screen time
WCST: The Wisconsin Card Sorting Test
IQ: Intelligence quotient
CC: Completed categories
SE: Shifting efficiency
NPE: Non-perseverative errors
FMS: Failure to maintain set
BMI: Body mass index
SD: Standard deviation
ANCOVA: Analysis of covariance
CI: Confidence interval
