# Supplementary material for: Association between the 24-hour movement guidelines and executive function among Chinese children
Source: BMC Public Health. 2022 May 20;22:1017. doi: 10.1186/s12889-022-13420-5 (PMC9121539; doi:10.1186/s12889-022-13420-5)
Supplement: Supplementary file 1 — Additional file 1: Table S1. Demographic characteristics of the analyzed subjects and sampling populations. Table S2. Performance of WCST with or without meeting recommendations for PA, ST, and sleep duration. Table S3. Associations between meeting the physical activity, screen time, and sleep duration recommendations and four dimensions of WCST in boys. [file 12889_2022_13420_MOESM1_ESM.docx]

**Table S1 Demographic characteristics of the analyzed subjects and sampling populations**

|  | Total Sample（N=637） | Included Sample（N=376） | *P* |
| --- | --- | --- | --- |
| Age | 9.20±1.63 | 9.17±1.61 | 0.234 |
| Sex |  |  | 0.807 |
| Boys | 323 (50.7) | 195 (51.9) |  |
| Girls | 314 (49.3) | 181 (48.1) |  |
| Paternal educational level |  |  | 0.051 |
| High school or below | 156 (25.2) | 91 (24.6) |  |
| Junior college | 144 (23.2) | 88 (23.8) |  |
| College or above | 320 (51.6) | 191 (51.6) |  |
| Maternal educational level |  |  | 0.428 |
| High school or below | 161 (26.0) | 87 (23.5) |  |
| Junior college | 155 (25.0) | 104 (28.1) |  |
| College or above | 304 (49.0) | 179 (48.4) |  |
| Household monthly income |  |  | 0.543 |
| ＜5000,CNY | 144 (23.3) | 92 (24.5) |  |
| 5000~7999, CNY | 154 (24.9) | 86 (22.9) |  |
| 8000~11999, CNY | 112 (181.) | 55 (14.6) |  |
| ≥12000, CNY | 132 (21.4) | 76 (20.2) |  |
| No answer | 76 (12.3) | 67 (17.8) |  |
| BMI | 17.32±6.20 | 17.33±7.53 | 0.865 |
| IQ score | 114.78±16.58 | 112.32±16.92 | 0.094 |

Data are presented as n (%), and *P*-value denotes Chi-Square test calculated the difference between three groups. Total sample refers to a sampling population who were asked to wear accelerometer; Included sample refers to participants in our analysis.

CNY,Chinese Yuan; BMI, Body Mass Index; IQ:Intelligence Quotient.

**Table S2 Performance of WCST with or without meeting recommendations for PA, ST, and sleep duration**

|  | PA | | *P*_1_ |  | ST | | *P*_2_ |  | Sleep duration | | *P*_3_ |
| --- | --- | --- | --- | --- | --- | --- | --- | --- | --- | --- | --- |
|  | Meet | Do not meet |  |  | Meet | Do not meet |  |  | Meet | Do not meet |  |
| CC | 5.37±1.03 | 4.81±1.59 | **0.009** |  | 4.93±1.55 | 4.77±1.38 | 0.432 |  | 5.02±1.42 | 4.59±1.72 | **0.015** |
| SE | 52.36±23.99 | 45.11±25.77 | **0.039** |  | 46.83±25.88 | 43.67±24.25 | 0.370 |  | 47.17±25.24 | 44.00±26.48 | 0.287 |
| NPE | 30.64±22.95 | 35.79±24.89 | 0.124 |  | 34.34±24.86 | 37.88±23.49 | 0.298 |  | 33.39±23.45 | 38.98±27.13 | **0.024** |
| FMS | 1.17±0.98 | 1.41±1.28 | 0.194 |  | 1.36±1.24 | 1.43±1.28 | 0.669 |  | 1.42±1.30 | 1.23±1.07 | 0.175 |

Data were presented as Mean ± SD. Independent sample t-test was used to analyze differences between the two groups.

WCST, the Wisconsin Card Sorting Test; SD, Standard deviation.PA, Physical activity; ST, Screen time;

CC, number of Completed Categories; SE, Shifting Efficiency; NPE, Non-Preservative Errors; FMS, Failure to Maintain Set.

**Table S3 Associations between meeting the physical activity, screen time, and sleep duration recommendations and four dimensions of WCST in boys**

|  |  | *β* (95% CI) |  |  |
| --- | --- | --- | --- | --- |
| Guidelines | CC | SE | NPE | FMS |
| Number of guidelines met | **0.360 (0.083,0.637) *** | 4.290 (-0.634,9.214) | **-5.460 (-10.165, -0.755) *** | 0.010 (-0.242,0.261) |
| PA |  |  |  |  |
| Meet | Reference | Reference | Reference | Reference |
| Do not meet | -0.443 (-0.904, 0.017) | -4.860(-13.015,3.294) | 3.589(-4.266,11.444) | 0.153(-0.262,0.569) |
| ST |  |  |  |  |
| Meet | Reference | Reference | Reference | Reference |
| Do not meet | -0.222(-0.782,0.337) | -4.881(-14.713,4.951) | 5.904(-3.536,15.345) | 0.050(-0.456,0.556) |
| Sleep Duration |  |  |  |  |
| Meet | Reference | Reference | Reference | Reference |
| Do not meet | -0.407(-0.884,0.070) | -3.667(-12.105,4.772) | 7.663(-0.388,15.715) | -0.225(-0.651,0.201) |
| PA＋ST |  |  |  |  |
| Meet | Reference | Reference | Reference | Reference |
| Do not meet | **-0.512(-0.987, -0.036) *** | -4.818(-13.263,3.628) | 3.976(-4.153,12.106) | 0.089(-0.342,0.520) |
| PA＋Sleep Duration |  |  |  |  |
| Meet | Reference | Reference | Reference | Reference |
| Do not meet | **-0.649(-1.155, -0.142) *** | **-10.881(-19.807, -1.955) *** | 6.876(-1.789,15.540) | 0.286 (-0.169,0.742) |
| ST＋Sleep Duration |  |  |  |  |
| Meet | Reference | Reference | Reference | Reference |
| Do not meet | -0.393(-0.826,0.041) | -5.964(-13.615,1.687) | 6.884(-0.452,14.219) | -0.067(-0.457,0.323) |
| All three recommendations |  |  |  |  |
| Meet | Reference | Reference | Reference | Reference |
| Do not meet | **-0.776(-1.311, -0.242) *** | **-11.728(-21.188, -2.269) *** | 7.897(-1.279,17.073) | 0.227(-0.257,0.710) |

Model was adjusted by age, paternal/maternal educational level, household monthly income, Body Mass Index and Intelligence Quotient.

Schools were ﬁtted as random eﬀects in models.

WCST, the Wisconsin Card Sorting Test; PA, Physical activity; ST, Screen time;

CC, number of Completed Categories; SE, Shifting Efficiency; NPE, Non-Preservative Errors; FMS, Failure to Maintain Set.

*:*P*<0.05 **:*P*<0.001

**Table S4 Associations between meeting the physical activity, screen time, and sleep duration recommendations and four dimensions of WCST in girls**

|  |  | *β* (95% CI) |  |  |
| --- | --- | --- | --- | --- |
| Guidelines | CC | SE | NPE | FMS |
| Number of guidelines met | 0.241 (-0.138,0.620) | 1.935(-3.957,7.827) | -1.712 (-7.489,4.064) | 0.129(-0.169,0.428) |
| PA |  |  |  |  |
| Meet | Reference | Reference | Reference | Reference |
| Do not meet | -0.365(-1.547,0.816) | -5.311(-23.615,12.993) | 3.736(-14.212,21.683) | 0.239(-0.688,1.165) |
| ST |  |  |  |  |
| Meet | Reference | Reference | Reference | Reference |
| Do not meet | 0.180(-0.478,0.838) | 2.128(-8.065,12.321) | -3.332(-12.313,6.650) | -0.069(-0.586,0.448) |
| Sleep Duration |  |  |  |  |
| Meet | Reference | Reference | Reference | Reference |
| Do not meet | **-0.612(-1.184, -0.040) *** | -4.870(-13.829,4.090) | 5.668(-3.099,14.435) | -0.304(-0.757,0.149) |
| PA＋ST |  |  |  |  |
| Meet | Reference | Reference | Reference | Reference |
| Do not meet | -0.784(-2.044,0.475) | -10.184(-19.718,9.351) | 11.817(-7.301,30.936) | 0.086(-0.906,1.078) |
| PA＋Sleep Duration |  |  |  |  |
| Meet | Reference | Reference | Reference | Reference |
| Do not meet | -0.297(-1.571,0.977) | -4.153(-13.890, 15.585) | 2.406(-16.944,21.757) | 0.213(-0.786,1.213) |
| ST＋Sleep Duration |  |  |  |  |
| Meet | Reference | Reference | Reference | Reference |
| Do not meet | -0.323(-0.850,0.204) | -2.794(-10.985,5.396) | 3.499(-4.520,11.519) | -0.221(-0.636,0.194) |
| All three recommendations |  |  |  |  |
| Meet | Reference | Reference | Reference | Reference |
| Do not meet | -0.779(-2.149,0.591) | -9.688(-30.941,11.566) | 11.716(-9.085,32.517) | 0.027(-1.051,1.106) |

Model was adjusted by age, paternal/maternal educational level, household monthly income, Body Mass Index and Intelligence Quotient.

Schools were ﬁtted as random eﬀects in models.

WCST, the Wisconsin Card Sorting Test; PA, Physical activity; ST, Screen time;

CC, number of Completed Categories; SE, Shifting Efficiency; NPE, Non-Preservative Errors; FMS, Failure to Maintain Set.

*:*P*<0.05 **:*P*<0.001
